# Assessment of blood and productive parameters in mid-lactation dairy cows fed different diets: replacement of corn silage with triticale silage

**DOI:** 10.5194/aab-65-223-2022

**Published:** 2022-05-31

**Authors:** Lorella Giuliotti, Maria Novella Benvenuti, Andrea Martini, Pier Attilio Accorsi, Claudia Lotti, Alice Cappucci, Giuseppe Conte

**Affiliations:** 1 Department of Veterinary Science, University of Pisa, Pisa 56124, Italy; 2 Department of Agricultural, Food and Forestry Systems, University of Firenze, Florence 50144, Italy; 3 Department of Veterinary Medical Sciences, University of Bologna, Ozzano dell'Emilia (BO) 40064, Italy; 4 Department of Agriculture, Food and Environment, University of Pisa, Pisa 56124, Italy

## Abstract

Corn crops require large amounts of resources that affect the environmental sustainability of dairy cow farming systems. The aim of the study was thus to investigate the effects of the replacement of corn silage (CS) with triticale silage (TS) by evaluating blood and productive parameters. The study lasted 7 weeks and involved two groups of 20 Italian Holstein Friesian dairy cows that were homogeneous in terms of parity ( 3±1.5 ), days in milk (DIM) ( 150±85.0 ), and daily milk production ( 26±4.6 kg).

Chemical analysis of feeds was carried out weekly. Dry-matter intake was estimated daily. At the beginning and end of the trial, haematological, metabolic, and immunological parameters were analysed. At the same, time body weight and body condition score were measured. Milk characteristics were also analysed weekly. Statistical analysis was performed by ANOVA on data of the second sampling, and a non-parametric test was performed to analyse BCS.

Regarding the haematological parameters in the two groups, only lymphocyte values were not in the normal range (2.86 and $2.50\times 10^{9}$ L for CS and TS, respectively). Metabolic parameters were in the normal range except for blood ureic nitrogen (BUN; 13.65 and 14.04 $\mathrm{mg}\;{\mathrm{dL}}^{-1}$ ), non-esterified fatty acids (NEFAs; 21.40 and 31.93 $µ\mathrm{mol}\;L^{-1}$ ), and Cl (91.99 and 93.50 $\mathrm{mmol}\;L^{-1}$ ). Hair cortisol was low (0.94 and 0.91 $\mathrm{pg}\;{\mathrm{mg}}^{-1}$ ), indicating the absence of stress signs, as confirmed by the results of other immunological parameters (serum lysozyme (SL), bactericidal activity (SBA), haptoglobin (HP), and oxygen free radicals (OFRs)).

Statistical differences were not found either for haematological or biochemical parameters. The total replacement of CS with TS did not affect milk yield and composition.

In conclusion, the replacement of CS by TS did not give rise to significant modifications in the parameters investigated and did not alter the health status of the animals, thus suggesting the feasibility of its introduction into the diet of mid-lactation dairy cows.

## 1 Introduction

Given the urgent need for the rational management of resources, greenhouse gases, and land use, some feed sources need to be replaced in the diet of dairy cows. This replacement is also necessary since the corn crops widely employed as silage for dairy cow feeding have a negative impact on resource requirements and pollution (Harper et al., 2017). Moreover, the use of corn in the feeding system of dairy cows needs to be converted due to the high likelihood of contamination from mycotoxin (Migliorati et al., 2017).

One possible replacement crop is triticale since it has a high yield, adapts well to a wide range of soil types and environments, and shows a low susceptibility to the common fungal diseases of cereals (Randhawa et al., 2015), together with a low water consumption (Cosentino et al., 2015).

Triticale is increasingly used for livestock due to its nutritional qualities. However, triticale starch shows a higher level of rumen digestibility than corn starch (93 % vs. 90 %) (Krieg et al., 2017), and it is advisable to avoid sudden changes in the diet, especially when it is added in high quantities (Myer and Lozano del Rio, 2004).

Health and productivity are closely connected. Blood metabolites are particularly informative regarding the animal's response to nutritional challenges (Satyendra and Om, 2016). Haematological and immunological parameters provide information on the animal's health condition, whereas hair cortisol highlights the activity of the hypothalamus pituitary adrenal axis (Comin et al., 2011) in response to stressors. Lastly, body weight (BW), body condition score (BCS), and milk quanti-qualitative characteristics represent the productive response.

Studies on the feasibility of the partial introduction of triticale in the diet of dairy cows in the transition period have been carried out (Mikula et al., 2011; Cosentino et al., 2015). The aim of this study was thus to assess the health of dairy cows following the total replacement of corn silage with triticale silage by evaluating the haematological, metabolic, immunological, and productive parameters in cows during mid-lactation.

## 2 Material and methods

The trial was carried out on the experimental dairy farm of the University of Pisa. Animals were handled as outlined in accordance with the guidelines of the European Union directive 2010/63/EU for animal experiments.

Forty multiparous healthy Italian Holstein Friesian dairy cows in mid-lactation were used in the experiment. No animal enrolled had experienced a change in social group or had been affected by any diseases in the period before the study.

The cows were managed in a free-stall system, milked twice daily, and fed a total mixed ration corresponding to the diet containing corn silage (CS), described in Table 1.

**Table 1:** Ingredients and chemical composition of the experimental diets (mean ± standard deviation).

	Diets			
	CS		TS	
Ingredients (% DM)	mean	SD	mean	SD
Alfalfa hay, first cut	8.78	0.45	8.37	0.48
Alfalfa hay, second cut	18.05	1.16	17.21	1.32
Concentrate ${}^{*}$	21.46	1.57	20.46	1.57
Cereal mix	25.85	1.96	24.65	1.76
Corn silage	25.85	1.96	0	
Triticale silage	0		29.30	1.41
Chemical composition (% of DM)				
Dry matter	48.9	2.0	46.6	1.7
Crude protein	13.1	1.1	13.2	1.3
Ether extract	3.3	0.4	3.3	1.4
Ash	5.6	0.7	5.3	0.4
Neutral detergent fibre (NDF)	36.8	2.9	35.3	2.4
Acid detergent fibre (ADF)	22.5	2.5	20.7	2.7
Acid detergent lignin (ADL)	14.3	1.4	14.6	1.7
Non-protein nitrogen (NPN)	1.4	0.1	1.5	0.1
Soluble carbohydrates	4.2	0.1	4.4	0.1
Starch	25.8	1.8	32.5	2.0
Metabolizable protein $g\;{\mathrm{kg}}^{-1}$	100.1	5.6	100.1	4.7
Titratable acidity ( $\mathrm{meq}\;g^{-1}$ )	7.2	0.2	6.4	0.1
Lactic acid (%DM)	4.8	0.4	5.1	0.1
Acetic acid (%DM)	2.5	0.2	4.9	0.3
Propionic acid (%DM)	0.2	0.0	0.4	0.0
Butyric acid (%DM)	0.0	0.0	0.0	0.0
Metabolizable energy $\mathrm{MJ}\;{\mathrm{kg}}^{-1}$	9.8	0.5	9.6	0.4
Net energy of lactation $\mathrm{MJ}\;{\mathrm{kg}}^{-1}$	5.9	0.2	5.9	0.2

${}^{*}$ Ingredient (%): corn meal 22.0; wheat middling 20.0; barley meal 17.5; soybean meal 17.0; extruded linseed 10.0; protein concentrate 5.0; beet pulp 5.0; vitamin–mineral mix 3.5.

At the beginning of the trial (T0), two groups of 20 cows were allocated randomly. The study lasted 7 weeks and involved two groups of 20 cows that were homogeneous in terms of parity ( 3±1.5 ), days in milk (DIM) ( 150±85.0 ), daily milk production ( 26 ± 4.6 kg), and BCS ( 3.01 ± 0.26 and 3.04±0.37 for CS and triticale silage (TS), respectively). The control group consumed the usual diet containing CS. The other group received a diet in which the corn silage had been totally replaced with TS. The trial ended after 7 weeks (T1). Water was offered ad libitum.

After harvest time (middle of May), triticale plants (Orval variety) were ensiled in silos, filled in 1 working day and sealed with polyethylene film. The inoculant, Pioneer 11A44^®^ containing LN 4637 *Lactobacillus buchneri* 100 %, was applied on forage. After 3 months of storage, silos were unloaded.

The ingredients and chemical composition of the experimental diets are reported in Table 1. At T0, the CS and TS groups were homogeneous in terms of parity ( 3±1.5 ), days in milk (DIM) ( 150±85.0 ), and daily milk production ( 26±4.6 kg).

Dry-matter intake (DMI) was estimated daily, by weighing the residue of the ration. The animals were fed in groups. Feed efficiency was calculated per dietary group by the ratio between kilograms of daily milk and kilograms of feed dry matter (DM) consumed. DMI and feed efficiency did not differ between the two groups.

Feed samples were collected every week for chemical analysis. The following analyses were performed: neutral detergent fibre (NDF), acid detergent fibre (ADF), and acid detergent lignin (ADL) (Van Soest et al., 1991), crude protein, ash, ether extract (AOAC, 2000), non-protein nitrogen (NPN), soluble nitrogen, nitrogen in NDF residues (NDFIP) and nitrogen in ADF residue (ADFIP) (Licitra et al., 1996), starch (McCleary et al., 1997), and soluble carbohydrates (Dubois et al., 1956).

Individual milk production was measured daily, and analyses of fat, protein, lactose, urea, and somatic cell count were performed weekly. Milk samples were analysed for fat, protein, and lactose by infrared analysis (Milkoscan 133 B; Italian Foss Electric, Padua, Italy). The somatic cell count (SCC) was calculated with a Fossomatic 215 cell counter (Foss Electric, 3400 Hillerod, Denmark). Data were transformed into linear scores according to Wiggans and Shook (1987).

Milk was sampled in the morning and was stored at 4 ${}^{\circ }C$ with a preservative (Bronopol solution, Lanxess, Corporation, Pittsburgh, PA) until the analysis.

At T0 and at T1, in the morning, immediately after feeding, blood samples were collected in quiet conditions from 10 cows randomly selected in each group by the farm veterinarian. Blood samples were taken from the jugular vein minimizing stress. Antiseptic gauze was applied to remove superficial dirt and debris. The jugular vein was visualized by applying pressure at the base of the jugular groove. The needle was firmly inserted into the skin and into the vein at a 20 ${}^{\circ }$ angle. Vacutainer tubes either with or without K3-ethylenediamine tetra-acetic acid (EDTA) as anticoagulant were employed.

The samples were kept in ice boxes and immediately sent to the laboratory.

The complete blood count was measured by the laboratory of the Veterinary Science Department of Pisa using a CELL-DYN 3500^®^ automated haematology analyser (Abbott, Minneapolis, USA). The following were analysed: red blood cells (RBCs), haematocrit (HCT), haemoglobin (HGB), mean corpuscular volume (MCV), mean corpuscular haemoglobin (MCH), reticulocytes (RETIC), white blood cell count (WBC), neutrophils (NEUs), lymphocytes (LYMs), monocytes (MONs), eosinophils (EOSs), basophils (BASs), and blood platelets (PLTs).

The following metabolic and immunological parameters were analysed by the Istituto Zooprofilattico Sperimentale Lazio e Toscana (IZSLT): alanine aminotransferase (ALT), aspartate aminotransferase (AST), beta-hydroxybutyric acid (BHBA), blood ureic nitrogen (BUN), non-esterified fatty acids (NEFAs), total proteins (TPs), creatinine (Creat), calcium (Ca), chlorine (Cl), phosphorus (P), potassium (K) and sodium (Na), and serum lysozyme (SL). Bactericidal activity (SBA) was determined according to validated procedures using a bacteriological assay (Osserman and Lawlor, 1966; Bonizzi et al., 1989; Ponti et al., 1989; Amadori et al., 2002). Haptoglobin (HP) and oxygen free radicals (OFRs) were monitored by a colorimetric method (Giuliotti et al., 2017).

At T0 and T1, hair samples were carefully cut from the tail switch using clippers and frozen at a temperature of -20 ${}^{\circ }C$ to prevent the presence of lice and were analysed according to Giuliotti et al. (2017).

Blood and hair samples were collected during daily routine activities in order not to disturb the animals.

At the same time, BW and BCS were recorded. BCS was recorded by the same observer using the 1–5 scale according to Ferguson et al. (1994), along with an increasing level of fattening.

Data were analysed with the following linear model, using JMP software (S.A.S., 2002):

$$y_{ij}=\mu +D_{i}+b(x_{ij}-x)+e_{ij},$$

where $y_{ij}$ is haematological, biochemical, and immunological parameters, milk yield and composition, and body weight; $D_{i}$ is the fixed effect of the i th diet (CS and TS); $T_{j}=\beta$ is the linear regression coefficient between $y_{ij}$ and $x_{ij}$ , $x_{ij}$ is the covariate value corresponding to $y_{ij}$ , x is the mean of $x_{ij}$ , and $e_{ij}$ is the residual error.

BCS was analysed by the Wilcoxon non-parametric test.

## 3 Results and discussion

BW (Table 2) and BCS did not differ significantly. BCS mean values were 3.01±0.29 and 3.04±0.31 for the CS and TS groups, respectively. McQueen and Fillmore (1991) reported similar BW results, while Mikula et al. (2011) reported similar BCS values.

**Table 2:** Haematological parameters of each sampling in the two experimental groups.

	Normal range	UM	CS	TS	SEM	P value
BW		kg	688.07	684.06	12.12	0.85
RBC	4.47–9.35	× 10 ${}^{12}$ L	5.99	6.23	0.11	0.15
HCT	22.5–39.9	%	28.15	28.72	0.84	0.64
HGB	7.4–12.8	$g\;{\mathrm{dL}}^{-1}$	9.58	10.08	0.19	0.08
MCV	40.4–56.4	fL	48.57	48.99	1.13	0.79
MCH	11.5–18.5	pg	16.12	16.22	0.21	0.72
RETIC	0–3.0	$K\;µL^{-1}$	1.44	1.42	0.30	0.96
WBC	2.71–17.76	$K\;µL^{-1}$	7.66	7.50	0.52	0.83
NEU	1.1–5	$\times 10^{9}$ L	3.78	3.79	0.50	0.99
LYM	3.2–6.8	$\times 10^{9}$ L	2.86 ↓	2.50 ↓	0.26	0.36
MON	0.1–0.8	$\times 10^{9}$ L	0.41	0.48	0.08	0.57
EOS	0.1–2.2	$\times 10^{9}$ L	0.50	0.55	0.14	0.82
BAS	0–0.2	$\times 10^{9}$ L	0.18	0.09	0.12	0.58
PLT	147–663	$K\;µL^{-1}$	348.30	363.50	43.41	0.81

Normal ranges were provided by the laboratory of the Department of Veterinary Science of Pisa. ↑ Values over the threshold of the normal range. ↓ Values under the threshold of the normal range. RBCs – red blood cells; HCT – haematocrit; HGB – haemoglobin; MCV – mean corpuscular volume; MCH – mean corpuscular haemoglobin; WBC – white blood cell count; NEUs – neutrophils; LYM – lymphocyte; MONs – monocytes; EOSs – eosinophils; BASs – basophils; PLTs – blood platelets; SEM – standard error of mean. UM (unit of measurements): kg (kilogram), % (percentage), $g\;{\mathrm{dL}}^{-1}$ (grams per decilitre), fL (femtolitre), pg (picogram), $K\;µL^{-1}$ (thousand per microlitre), L (litre).

No significant differences were detected for the haematological parameters (Table 2). Almost all the values fell within the range of reference for healthy dairy cows, with only LYM not in this range. A moderate lymphopenia is a common finding in stressed animals (Gleeson et al., 2007); however, in the present survey this condition was not confirmed by the outcome of the other parameters such as hair cortisol level and other immunological parameters. Lymphopenia has been described during acute viral or bacterial infections (Jones and Allison, 2007); however, the cows in our study did not present these conditions. In any case, lymphopenia is not attributable to the diet.

Regarding the metabolic parameters (Table 3), no significant differences were found. This was also confirmed in a study by Gorelik et al. (2020), in which the introduction of triticale in the diet of cattle did not affect any of the examined metabolic parameters (AST, TP, Ca, P).

**Table 3:** Biochemical and immunological parameters of each sampling in the two experimental groups.

Parameter	Normal range	UM	CS	TS	SEM	P value
ALT	14–38	$U\;L^{-1}$	34.58	36.31	1.61	0.46
AST	60–118	$U\;L^{-1}$	84.86	93.04	4.92	0.24
BHBA	<10.5	$\mathrm{mg}\;{\mathrm{dL}}^{-1}$	9.11	7.48	0.99	0.28
BUN	20–30	$\mathrm{mg}\;{\mathrm{dL}}^{-1}$	13.65 ↓	14.04 ↓	1.03	0.79
NEFA	89–618	$µ\mathrm{mol}\;L^{-1}$	21.40 ↓	31.93 ↓	4.08	0.11
TP	5.7–8.1	$g\;{\mathrm{dL}}^{-1}$	7.72	7.76	0.17	0.86
Creat	1–2.7	$\mathrm{mg}\;{\mathrm{dL}}^{-1}$	1.01	1.04	0.02	0.51
Ca	8–10.5	$\mathrm{mg}\;{\mathrm{dL}}^{-1}$	9.30	9.43	0.16	0.59
Cl	97–111	$\mathrm{mmol}\;L^{-1}$	91.99 ↓	93.50 ↓	0.41	0.12
P	4–7	$\mathrm{mg}\;{\mathrm{dL}}^{-1}$	6.14	6.19	0.31	0.91
K	3.9–5.8	$\mathrm{mmol}\;L^{-1}$	4.14	4.25	0.12	0.53
Na	135–151	$\mathrm{mmol}\;L^{-1}$	136.06	134.94	0.80	0.35
SL	1–3	$µg\;{\mathrm{mL}}^{-1}$	1.18	0.78 ↓	0.21	0.20
HP	0.0–0.5	$\mathrm{mg}\;{\mathrm{mL}}^{-1}$	0.35	0.13	0.08	0.09
OFR ${}^{*}$		U.carr ${}^{**}$	34.01	29.99	2.82	0.33
SBA ${}^{*}$	>90	%	90.53	89.47 ↓	0.86	0.39
Hair cortisol		$\mathrm{pg}\;{\mathrm{mg}}^{-1}$	0.94	0.91	0.15	0.89

Normal ranges were provided by the laboratory of IZSLT. SEM stands for standard error of the mean. ${}^{*}$ No laboratory reference range is available. ${}^{**}$ U. Carr. is an arbitrary unit; 1 U. Carr. is equivalent to 0.08 mg of $H_{2}O_{2}$ per mg dl ${}^{-1}$ . ↑ Values over the threshold of the normal range. ↓ Values under the threshold of the normal range. ALT – alanine aminotransferase; AST – aspartate aminotransferase; BHBA (beta-hydroxybutyric acid; BUN – nitrogen ureic; NEFAs – non-esterified fatty acids; TPs – total proteins; Creat – creatinine; Ca – calcium; Cl – chlorine; P – phosphorus; K – potassium; Na – sodium; SL – serum lysozyme; SBA – bactericidal activity; HP – haptoglobin; OFRs – oxygen free radicals; and hair cortisol. UM (unit of measurements): $U\;L^{-1}$ (unit per litre), $\mathrm{mg}\;{\mathrm{dL}}^{-1}$ (milligram per decilitre), $µ\mathrm{mol}\;L^{-1}$ (micromole per litre), $g\;{\mathrm{dL}}^{-1}$ (gram per decilitre), $\mathrm{mmol}\;L^{-1}$ (millimole per litre), $µg\;{\mathrm{mL}}^{-1}$ (microgram per millilitre); U.carr (Carratelli unit); % (percentage); $\mathrm{pg}\;{\mathrm{mg}}^{-1}$ (picogram per milligram).

Only BUN, NEFA, and Cl values were not in the normal range in the two groups.

NEFA together with BHBA is considered important in evaluating the negative energy balance in dairy cows. In our case, this was not a problem because the NEFA values were low and BHBA was in the normal range.

High plasma NEFA levels with other parameters such as BHBA and OFR (Herdt, 2000) could be indices of inflammation. In our study, no critical situation regarding these parameters was shown (Table 3) (Giuliotti et al., 2017; Benvenuti et al., 2018). In fact, although NEFA was far below the normal threshold, Cozzi et al. (2011) reported that in mid-lactation NEFA concentration decreases when the energy balance turns positive; in any case, Oetzel (2004) reported that low NEFA concentrations are not biologically important.

BUN values were also found to be under the threshold. BUN is strictly related to protein because the urea derived from nitrogen deamination of amino acids is not utilized for milk synthesis (Kohn, 2007). Low BUN levels can sometimes be explained by a deficient protein and energy intake (Lee et al., 1978); however, in our trial the diets were adequately formulated (Table 1) for low-producing dairy cows. This occurrence has been noticed by Nozad et al. (2012), who, in a study on low- and high-producing cow (average daily milk production 29 and 32 $\mathrm{kg}\;d^{-1}$ ), found the lowest values of BUN in the cow with the lowest milk yield.

Most minerals are regulated in the body through homeostatic processes and their concentrations are rarely due to an insufficient supply in the diet (Van Saun, 2008). In our study, Cl values were slightly under the normal range in the two groups. Skrzypczak et al. (2014) reported a chlorine concentration in the blood ranging from 93 to 107 $\mathrm{mmol}\;L^{-1}$ in healthy cows. The same authors reported that the concentration of Cl is associated with the Na concentration, which however was in the normal range. The other minerals (Ca and K) fell within the normal range, thus not indicating a health impairment.

SL, SBA, and HP represent a nonspecific cellular immune response. Our results regarding the first two parameters indicated a slightly altered immune response in the experimental group. It is well known that the functionality of the immune system can be influenced by diet (Dänicke et al., 2018). An alteration in these parameters may indicate inappropriate feed management in addition to inadequate hygienic and sanitary conditions of the herd (Bonizzi et al., 2003). As SL is involved in the immune system, it is one of the most predictive parameters of disease. Variations in SL levels have been found in response to inflammation or metabolic stress-related conditions in early lactation (Trevisi et al., 2012). Bonizzi et al. (2003) also reported a decrease in SL in cows during the transition period. Regarding SBA, some authors have reported that values around 90 % represent a sign of altered physiological conditions, thus indicating a predisposition to developing diseases conditioned by stressful events (Amadori et al., 2002). Despite all these considerations, as statistical differences were not found between the two groups, the low SL and SBA values were not ascribable to the triticale silage.

Investigating hair cortisol is important due to its relationship with chronic stress (Accorsi et al., 2008). The values that we found for hair cortisol were lower than those found in the literature. Del Rosario et al. (2011) reported hair cortisol concentrations equal to 12.15±1.85 $\mathrm{pg}\;{\mathrm{mg}}^{-1}$ in 2-year-old cows, and Burnett et al. (2014) found a cortisol level equal to 11.0±1.2 $\mathrm{pg}\;{\mathrm{mg}}^{-1}$ in lactating dairy cows. Benvenuti et al. (2018) observed higher values in dairy cows reared in an open-stall system.

The total replacement of CS with TS did not affect milk yield and composition (Table 4). Our results on milk yield confirmed the study by Cosentino et al. (2015) and Migliorati et al. (2017), who found no differences when replacing corn with triticale and barley silage, respectively. On the other hand, Harper et al. (2017) reported a milk yield decrease as a consequence of a partial substitution of corn silage with triticale silage. This contrasting result could be due to the sensitivity of dairy cows to modifications in the diet in high-yielding dairy cows (Enemark, 2008).

**Table 4:** Milk yield and milk composition.

Diet	CS	TS	SEM	P value	UM
Milk yield	26.54	25.96	1.40	0.43	$\mathrm{kg}\;d^{-1}$
Fat	3.62	3.83	0.15	0.31	%
Protein	3.34	3.29	0.07	0.62	%
Casein	2.83	2.86	0.06	0.77	%
Lactose	4.89	4.85	0.04	0.89	%
SCC	5.19	5.32	0.11	0.33	log10

SCC, somatic cell count.

The introduction of triticale in the diet did not affect the milk composition, in agreement with other authors (McQueen and Fillmore, 1991; Mikula et al., 2011; Harper, 2017).

## 4 Conclusions

Our results showed that in our experimental conditions, the complete substitution of corn silage with triticale silage did not induce differences in the investigated blood parameters and thus did not impair the health of the animals. In fact, hair cortisol, which is an indicator of chronic stress, was not influenced by the change in diet and showed a low concentration. In conclusion, since most of the alterations observed were not related to the diet and the productive parameters were unchanged, triticale silage would seem to be a valid replacement for corn when used in non-high-producing dairy cows.

## Data Availability

The datasets generated and/or analysed during the current study are available from the corresponding author on reasonable request.

## References

[bib1.bib1] Accorsi PA, Carloni E, Valsecchi P, Viggiani R, Gamberoni M, Tamanini C, Seren E (2008). Cortisol determination in hair and faeces from domestic cats and dogs. Gen Comp Endocr.

[bib1.bib2] Amadori M, Archetti IL, Mondelli MM, Fazia M (2002). La valutazione del benessere animale. Quaderni Fondazione Iniziative Zooprofilattiche E Zootecniche.

[bib1.bib3] AOAC, Association of Official Analytical Chemists (2000). Official Methods of Analysis.

[bib1.bib4] Benvenuti MN, Giuliotti L, Lotti C, Accorsi PA, Petrulli C, Martini A (2018). Welfare parameters in dairy cows reared in tie-stall and open-stall farming systems: pilot study. J Hell Vet Med Soc.

[bib1.bib5] Bonizzi L, Amadori M, Melegari M, Ponti W, Ceccarelli A (1989). Characterization of some parameters of non-specific immunity in dairy cattle (I). J Vet Med B.

[bib1.bib6] Bonizzi L, Menandro ML, Pasotto D, Lauzi S (2003). Transition Cow: Non-specific Immune Response. Vet Res Commun.

[bib1.bib7] Burnett TA, Madureira AML, Silper BF, Nadalin A, Tahmasbi A, Veira DM, Cerri RLA (2014). Factors affecting hair cortisol concentrations in lactating dairy cows. J Dairy Sci.

[bib1.bib8] Comin A, Prandi A, Peric T, Corazzin M, Dovier S, Bovolenta S (2011). Hair cortisol levels in dairy cows from winter housing to summer highland grazing. Livest Sci.

[bib1.bib9] Cosentino C, Adduci F, Musto M, Paolino R, Freschi P, Pecora G, D'Adamo C, Valentini V (2015). Low vs. high “Water Footprint Assessment” diet in milk production: A comparison between triticale and corn silage based diets. Emir J Food Agr.

[bib1.bib10] Cozzi G, Ravarotto L, Gottardo F, Stefani AL, Contiero B, Moro L, Brscic M, Dalvit P (2011). Reference values for blood parameters in Holstein dairy cows: Effects of parity, stage of lactation, and season of production. J Dairy Sci.

[bib1.bib11] Dänicke S, Meyer U, Kersten S, Frahm J (2018). Animal models to study the impact of nutrition on the immune system of the transition cow. Res Vet Sci.

[bib1.bib12] Del Rosario González-De-La-Vara M, Valdez RA, Lemus-Ramirez V, Vázquez-Chagoyán JC, Villa-Godoy A, Romano MC (2011). Effects of adrenocorticotropic hormone challenge and age on hair cortisol concentrations in dairy cattle. Can J Vet Res.

[bib1.bib13] Dubois M, Gilles KA, Hamilton JK, Rebers PA, Smith F (1956). Colorimetric method for determination of sugars and related substances. Anal Chem.

[bib1.bib14] Enemark JMD (2008). The monitoring, prevention and treatment of sub-acute ruminal acidosis (SARA): a review. Vet J.

[bib1.bib15] Ferguson JD, Galligan DT, Thomsen N (1994). Principal descriptors of body condition score in Holstein cows. J Dairy Sci.

[bib1.bib16] Giuliotti L, Benvenuti MN, Lai O, Accorsi PA, Rizzuto M, Lotti C, Petrulli CA, Martini A (2017). Welfare parameters in dairy cows reared in tie-stall and open-stall housing systems. Anim Sci Pap Rep.

[bib1.bib17] Gleeson DE, O'Brien B, Boyle L, Earley B (2007). Effect of milking frequency and nutritional level on aspects of the health and welfare of dairy cows. Animal.

[bib1.bib18] Gorelik OV, Gafner VD, Nesterenko AA, Dolmatova IA, Safronov SL, Ioan OGA (2020). Effect of triticale grain in feeding of dairy cows on their milk production and physiological state.

[bib1.bib19] Harper MT, Oh J, Giallongo F, Roth GW, Hristov AN (2017). Inclusion of wheat and triticale silage in the diet of lactating dairy cows. J Dairy Sci.

[bib1.bib20] Herdt TH (2000). Ruminant adaptation to negative energy balance: Influences on the etiology of ketosis and fatty liver. Vet Clin N Am-Food A.

[bib1.bib21] Jones ML, Allison RW (2007). Evaluation of the ruminant complete blood cell count. Vet Clin N Am-Food A.

[bib1.bib22] Kohn R (2007). Use of milk or blood urea nitrogen to identify feed management inefficiencies and estimate nitrogen excretion by dairy cattle and other animals.

[bib1.bib23] Krieg J, Seifried N, Steingass H, Rodehutscord M (2017). In situ and in vitro ruminal starch degradation of grains from different rye, triticale and barley genotypes. Animal.

[bib1.bib24] Lee AJ, Twardock AR, Bubar RH, Hall JE, Davis CL (1978). Blood metabolic profiles: their use and relation to nutritional status of dairy cows. J Dairy Sci.

[bib1.bib25] Licitra G, Hernandez TM, Van Soest PJ (1996). Standardization of procedure for nitrogen fractionation of ruminant feed. Anim Feed Sci Tech.

[bib1.bib26] McCleary BV, Gibson TS, Mugford DC (1997). Measurement of total starch in cereal products by amyloglucosidase-
α
-amylase method: collaborative study. J AOAC Int.

[bib1.bib27] McQueen RE, Fillmore AE (1991). Effects of triticale (cv. Beaguelita) and barley-based concentrates on feed intake and milk yield by dairy cows. Can J Anim Sci.

[bib1.bib28] Migliorati L, Boselli L, Pirlo G, Moschini M, Masoero F (2017). Corn silage replacement with barley silage in dairy cows' diet does not change milk quality, cheese quality and yield. J Sci Food Agr.

[bib1.bib29] Mikula R, Nowak W, Jaskowski JM, Mackowiak P (2011). Effects of different starch sources on metabolic profile, production and fertility parameters in dairy cows. Pol J Vet Sci.

[bib1.bib30] Myer R, Lozano Del Rio AJ, Mergoum M, Gómez-Macpherson H (2004). Triticale improvement and production, FAO Plant Production and Protection.

[bib1.bib31] Nozad S, Ramin AG, Moghadam G, Asri-Rezaei S, Babapour A, Ramin S (2012). Relationship between blood urea, protein, creatinine, triglycerides and macro-mineral concentrations with the quality and quantity of milk in dairy Holstein cows. Vet Res Forum.

[bib1.bib32] Oetzel GR (2004). Monitoring and testing dairy herds for metabolic disease. Vet Clin N Am-Food A.

[bib1.bib33] Osserman EF, Lawlor DP (1966). Serum and urinary lysozyme (muramidase) in monocytic and monomyelocytic leukemia. J Exp Med.

[bib1.bib34] Ponti W, Amadori M, Agnolotti F, Ionizzi L, Peri E, Caldora C (1989). Characterization of some parameters of non-specific immunity in beef cattle. J Vet Med B.

[bib1.bib35] Randhawa HS, Bona L, Graf RJ, Eudes F (2015). Triticale.

[bib1.bib36] SAS (2002). JMP User's Guide ver. 5.0.

[bib1.bib37] Satyendra KM, Om PS (2016). Blood Biochemical Profile and Nutritional Status of Dairy Cows under Field Conditions. J Anim Res.

[bib1.bib38] Skrzypczak A, Ska AK, Ski US, Jarosz A (2014). Sodium, potassium and chloride homeostasis in cows during pregnancy and first months of lactation. Acta Biol Cracov Zoo.

[bib1.bib39] Trevisi E, Amadori M, Cogrossi S, Razzuoli E, Bertoni G (2012). Metabolic stress and inflammatory response in high-yielding, periparturient dairy cows. Res Vet Sci.

[bib1.bib40] Van Saun RJ (2008). Metabolic Profiling of Transition Cows: Can We Predict Impending Problems?.

[bib1.bib41] Van Soest PJ, Robertson JB, Lewis BA (1991). Methods of dietary fiber, neutral detergent fiber, and nonstarch polysaccharides in relation to animal nutrition. J Dairy Sci.

[bib1.bib42] Wiggans GR, Shook G (1987). A lactation measure of somatic cell count. J Dairy Sci.

